# Predictors of Hospitalisation in Patients with Rheumatic Disease and COVID-19 in Ireland: data from the COVID-19 Global Rheumatology Alliance registry

**DOI:** 10.1093/rap/rkab031

**Published:** 2021-05-13

**Authors:** Richard Conway, Elena Nikiphorou, Christiana A Demetriou, Candice Low, Kelly Leamy, John G Ryan, Ronan Kavanagh, Alexander D Fraser, John J Carey, Paul O’Connell, Rachael M Flood, Ronan H Mullan, David J Kane, Philip C Robinson, Jean W Liew, Rebecca Grainger, Geraldine M McCarthy, for the COVID-19 Global Rheumatology Alliance

**Affiliations:** 1 Department of Rheumatology, St. James’s Hospital, James Street, Dublin, Ireland; 2 Trinity College Dublin, Ireland; 3 Department of Rheumatology, King’s College Hospital, London, UK; 4 Centre for Rheumatic Diseases, King’s College London, London, UK; 5 Department of Primary Care and Population Health, University of Nicosia Medical School, Nicosia, Cyprus; 6 Department of Rheumatology, St. Vincent’s University Hospital, Elm Park, Dublin, Ireland; 7 Department of Rheumatology, Mater Misericordiae Hospital, Dublin, Ireland; 8 Department of Rheumatology, Cork University Hospital, Cork, Ireland; 9 Galway Clinic, Galway, Ireland; 10 Department of Rheumatology, University Hospitals Limerick, Limerick, Ireland; 11 Graduate Entry Medical School, University of Limerick, Limerick, Ireland; 12 Department of Rheumatology, Galway University Hospitals, Galway, Ireland; 13 Medicine, National University of Ireland Galway, Galway, Ireland; 14 Department of Rheumatology, Beaumont Hospital, Dublin, Ireland; 15 RCSI, Dublin, Ireland; 16 Department of Rheumatology, Tallaght University Hospital, Dublin, Ireland; 17 Trinity College Dublin, Dublin, Ireland; 18 University of Queensland Faculty of Medicine, Brisbane, QLD, Australia; 19 Section of Rheumatology, Boston University School of Medicine, Boston, Massachusetts, USA; 20 Department of Medicine, University of Otago, Wellington, New Zealand

**Keywords:** Rheumatic disease, COVID-19, biologics, hospitalisation

## Abstract

**Objectives:**

Given the limited data regarding the risk of hospitalisation in patients with rheumatic disease and COVID-19 in Ireland we used the COVID-19 Global Rheumatology Alliance (GRA) registry data to study outcomes and their predictors. The primary objective was to explore potential predictors of hospitalisation.

**Methods:**

We examined data on patients and their disease-related characteristics entered into the COVID-19 GRA provider registry from Ireland (24^th^ March 2020 to 31^st^ August 2020). Multivariable logistic regression was used to assess the association of demographic and clinical characteristics with hospitalisation.

**Results:**

Of 105 patients, 47 (45.6%) were hospitalised and 10 (9.5%) died. Multivariable logistic regression analysis showed age (OR = 1.06, 95%CI 1.01 to 1.10), number of comorbidities (OR = 1.93, 95%CI 1.11 to 3.35), and glucocorticoid use (OR = 15.01, 95%CI 1.77 to 127.16) were significantly associated with hospitalisation. A diagnosis of inflammatory arthritis was associated with a lower odds of hospitalisation (OR = 0.09, 95%CI 0.02 to 0.32).

**Conclusion:**

Increasing age, comorbidity burden, and glucocorticoid use were associated with hospitalisation, while a diagnosis of inflammatory arthritis was associated with lower odds of hospitalization.

**Key messages**

1. Increasing age, comorbidity burden, and glucocorticoid use are associated with higher odds hospitalisation.

2. A diagnosis of inflammatory arthritis is associated with lower odds of hospitalization.

3. Insights from this study can help direct local strategies for the management of COVID-19 in this patient group.

## Introduction

Infection with the severe acute respiratory syndrome coronavirus 2 (SARS-CoV-2) and the subsequent Coronavirus disease 2019 (COVID-19) has caused considerable morbidity and mortality worldwide. Patients with rheumatic diseases are generally at increased risk of infection due to both their disease and the associated comorbidities, and immunomodulatory or suppressive treatments (1). Whether this applies to SARS-CoV-2 infection and the magnitude of any such risk remains unclear.

The COVID-19 Global Rheumatology Alliance (C19-GRA) aims to address the knowledge gap about COVID-19 for patients with rheumatic disease (2). The C19-GRA has reported data on the first 600 cases in its physician-reported case registry (3). In these patients' glucocorticoid doses of prednisolone-equivalent ≥10mg/day was associated with increased risk of hospitalisation whereas tumour necrosis factor alpha inhibitors (TNFi) were associated with a decreased risk of hospitalisation (3). Overall, as with the general population, the greatest risk of negative outcomes was associated with increasing age and comorbidities.

Geographical variations in COVID-19 outcomes have been reported (4-8). Our aim was to report the outcomes and their determinants in patients in Ireland with rheumatic disease who developed COVID-19.

## Methods

### COVID-19 Global Rheumatology Alliance

Data regarding individuals with rheumatic disease who develop COVID-19 are entered into one of two parallel international data portals hosted in the United States and United Kingdom. Details of the C19-GRA registries have been published previously (2, 3, 9).

### Data collection

Patients in this study were entered into the C19-GRA provider registry from 24th March 2020 to 31st August 2020.

Data collected on baseline rheumatic disease status including demographic and clinical variables were; age, sex, smoking status, rheumatic disease diagnosis, disease activity and comorbidities. Medications were categorised as previously described (Supplementary Data S1, available at *Rheumatology Advances in Practice* online) (3). Rheumatic diseases were categorised as 1) inflammatory arthritis, 2) gout, 3) vasculitis, connective tissue diseases, and others (Supplementary Data S2, available at *Rheumatology Advances in Practice* online). Data collected regarding COVID-19 infection included method of diagnosis, place of diagnosis, COVID-19 symptoms, and outcomes of COVID-19 disease including hospitalisation, ventilation, and death.

Continuous variables were reported as median (IQR). Categorical variables were reported as number and percentage (%). In univariable analyses, differences according to hospitalisation status and mortality were compared using Chi-squared tests or Fisher's exact tests, as appropriate, for categorical variables and Mann-Whitney U tests for continuous variables. The independent associations between demographic and disease-specific features with the odds of COVID-19 hospitalisation were estimated using first univariable and then multivariable-adjusted logistic regression and reported as odds ratios (OR) and 95% confidence intervals (CIs). We constructed two multivariable models; the first model included all variables with significant effect sizes ($p<0.05$) in the univariable logistic regression analyses; the second model was the most parsimonious model including age, gender, and those variables retaining statistical significance. We did not proceed to logistic regression analyses with mortality as the outcome due to the small number of deaths.

This study was approved by the Irish National Research Ethics Committee for COVID-19 (20-NREC-COV-010). The committee waived the need for written informed patient consent as the data were fully anonymised.

**Results**

The characteristics of the 105 included patients are shown in Table 1. Median age was 59 years and 61% were female. The most common rheumatic disease grouping was inflammatory arthritis (61/105, 41.9%) followed by connective tissue disease and others (25/105, 23.8%), and gout (21/105, 20%). Comorbidities were present in almost two-thirds of patients (67/105, 63.8%); the two most common were hypertension (32/105, 30.5%) and cardiovascular disease (26/105, 24.8%). Before COVID-19 diagnosis, glucocorticoids were prescribed to 15/105 (14.3%), csDMARD monotherapy to 33/105 (31.4%) and bDMARDs or tsDMARDs to 37/105 (35.2%). Almost half of reported cases were hospitalised (47/105, 45.6%) and 10 (9.5%) died.

When compared to patients not hospitalized, hospitalised patients were older, with a median age of 75 years compared to 51 years ($p<0.001$) (Table 2). A higher proportion of patients with gout were hospitalised (100%, $p<0.001$), while a lower proportion of patients with inflammatory arthritis were hospitalised (25%, $p<0.001$). Patients with no comorbidities had a lower frequency of hospitalisation (6/47, 15.8%, $p<0.001$), and hospitalized patients had a higher median number of comorbidities (median 2 vs 0, $p<0.001$). Hospitalisation was more common in patients on glucocorticoids (85.7%, $p=0.001$), with patients on ≥10mg prednisolone equivalent all hospitalized (100%, $p=0.008$).

Hospitalisation was less frequent among those prescribed csDMARD monotherapy (31.2%, p=0.049) and those prescribed bDMARDs or tsDMARDs either alone or in combination with csDMARDs (24.3%, p=0.001).

In the multivariable logistic regression model including all variables with significant effects estimates in univariable logistic regression analyses, glucocorticoid use was associated with a higher odds of hospitalisation (OR=18.14, 95%CI 1.13 to 290.81, p=0.041) (Table 2). A diagnosis of inflammatory arthritis was associated with a lower odds of hospitalisation (OR=0.18, 95%CI 0.02 to 0.95, p=0.044). In the most parsimonious multivariable logistic regression model, age (OR=1.06, 95%CI 1.01 to 1.10, p=0.01), number of comorbidities (OR=1.93, 95%CI 1.11 to 3.35, p=0.02), and glucocorticoid use (OR=15.01, 95%CI 1.77 to 127.16, p=0.013) were associated with an increased odds of hospitalisation. A diagnosis of inflammatory arthritis was associated with a lower odds of hospitalisation (OR=0.09, 95%CI 0.02 to 0.32, p<0.001). csDMARDs and b/tsDMARDs were not associated with hospitalisation in the multivariable analysis.

In unadjusted analyses deceased patients were older, median 80.5 years vs 58 years (p=0.001). No patients with no comorbidities died of COVID-19 (0%, p=0.013), while patients who died had a median of 3.5 comorbidities (median 3.5 vs 0, p<0.001) (Supplementary Table S1, available at *Rheumatology Advances in Practice* online). Specific comorbidities associated with mortality included cardiovascular disease (23.1%, p=0.014), diabetes mellitus (36.4%, p=0.01), hypertension (21.9%, p=0.008), renal disease (40%, p=0.007) and interstitial lung disease (66.7%, p=0.023).

**Discussion**

This is the largest report of people with rheumatic disease and COVID-19 from Ireland. We identified factors associated with higher odds of hospitalisation including increasing age, number of comorbidities, a diagnosis of gout, and baseline glucocorticoid use. A diagnosis of inflammatory arthritis was protectively associated with hospitalisation.

Our finding of the association of increasing age and glucocorticoid use with hospitalisation are consistent with the findings from C19-GRA (3). This highlights that glucocorticoid use should be limited in favour of longer-term disease modifying therapies. The C19-GRA reported bDMARDs as being associated with a reduced risk of hospitalisation, which has some biologic plausibility due to the potential for reduction of the hyperinflammatory state seen in severe COVID-19 (3, 10, 11). Several bDMARDs have been proposed as treatment for severe COVID-19, however, results of many controlled trials have been disappointing (12, 13). The RECOVERY and REMAP-CAP trials have demonstrated evidence of modest benefit from the use of the interleukin-6 receptor antagonists tocilizumab and sarilumab in COVID-19 (14, 15). In our population the apparent benefit of both csDMARDs and bDMARDs in the univariable analysis disappeared after controlling for underlying diagnostic group. These agents, and particularly bDMARDs, are more likely to be used in patients with a diagnosis of inflammatory arthritis and the reduced risk of hospitalization with these agents may represent confounding by indication. The results of ongoing randomised controlled trials of other bDMARDs in COVID-19 will provide clarity.

Our study has several limitations. Although the low number of cases of people with rheumatic diseases and COVID-19 in Ireland entered into the C-19 GRA registry limits the power of our study, this study explores associations between patient and disease characteristics and outcomes in Ireland. Potential overfitting of multivariable models may occur with small sample sizes and is one reason we did not proceed to multivariable analysis for mortality. Once further cases are entered into the GRA registries we will be able to conduct more detailed analyses in the future. As a physician entered registry, the C19-GRA is limited by selection bias with more severe cases likely to be entered, which likely explains why 100% of gout cases were hospitalized. As there is no denominator population for these data, no inferences can be drawn about the incidence of COVID-

19 in patients with rheumatic diseases. The C19-GRA is restricted to patients who have both rheumatic diseases and COVID-19, therefore no comparisons can be made between this group and the general rheumatic disease population nor to patients without rheumatic disease who develop COVID-19. Our finding of gout as a risk factor for hospitalisation, while highly significant as all gout cases were hospitalised, may reflect selection bias and requires future evaluation. Gout is usually managed in primary care in Ireland and since the C19-GRA is a rheumatologist-entered registry these cases could be less likely to be entered. Patients with rheumatic diseases may have behaved differently with regard to risk behaviour during COVID-19. In Ireland, a strict national lockdown was in place for a large part of the study period (including when most cases were reported). Individuals judged to be at high risk at the start of the pandemic, including many rheumatic diseases, were specifically advised to stay at home as much as possible and avoid social interactions (16).

In conclusion our findings confirm that increasing age, comorbidity burden, and glucocorticoid use are associated with hospitalisation with COVID-19 in patients with rheumatic diseases. An underlying diagnosis of inflammatory arthritis appears to be associated with lower risk of hospitalisation. The previously reported beneficial effects of bDMARDs may be mediated by underlying diagnosis rather than a specific effect.

**Acknowledgements**

The authors would like to thank all rheumatology providers who entered data into the registry. A list of COVID-19 Global Rheumatology Alliance members can be found in the supplementary material, available at *Rheumatology Advances in Practice* online.

RC, CL, KL, JGR, RK, ADF, JJC, POC, RMF, RHM, DJK, and GMM contributed to data collection, data quality control, data analysis and interpretation. They drafted, and revised, the manuscript critically for important intellectual content and gave final approval of the version to be published. EN and CAD contributed to the analysis and interpretation of the data. They drafted, and revised, the manuscript critically for important intellectual content and gave final approval of the version to be published. PCR, JWL and RG directed the work and contributed to the analysis and interpretation of the data. They drafted, and revised, the manuscript critically for important intellectual content and gave final approval of the version to be published.

**Funding:** No specific funding was received from any funding bodies in the public, commercial or not-for-profit sectors to carry out the work described in this manuscript.

**Disclosure statement:** R.C. is a member of the speakers' bureau for Janssen, Roche, Sanofi and Abbvie. E.N. is a member of the speakers' bureau for AbbVie, Eli-Lilly, Gilead, Celltrion, Pfizer and Sanofi. P.R. is a member of the speakers' bureau for UCB, Roche, Pfizer, Gilead, Janssen, Novartis, Eli Lilly and Abbvie, and has received grant/research support from Abbvie, UCB, Novartis, Janssen and Pfizer. J.L. has received grant/research support from Pfizer. R.G. is a member of the speakers' bureau for Pfizer, Cornerstones, Janssen, Novartis and Abbvie. The other authors have declared no conflicts of interest.

**Data availability statement:** Request for access to data from the registry should be made to the Data Access and Sharing Committee of the COVID-19 Global Rheumatology Alliance. The data underlying this article are available on reasonable request to the corresponding author.

**Table 1. Characteristics of patients with rheumatic disease diagnosed with COVID-19; all participants and by hospitalisation status.**

| | All Participants (n=105) | Not hospitalised (n=56) | Hospitalised (n=47) | |
|---|---|---|---|---|
| | N(%) | N(%) | N(%) | p-value[∞] |
| Gender | | | | |
| Female | 64 (61.0) | 39 (61.9) | 24 (38.10) | 0.054 |
| Male | 41 (39.0) | 17 (42.5) | 23 (57.5) | |
| Age (years) | | | | |
| 18-29 | 3 (2.9) | 2 (66.7) | 1 (33.3) | <0.001[a] |
| 30-49 | 25 (23.8) | 21 (84.0) | 4 (16.0) | |
| 50-65 | 32 (30.5) | 22 (71.0) | 9 (29.0) | |
| >65 | 45 (42.9) | 11 (25.0) | 33 (75.0) | |
| Median (IQR) | 59 (27) | 51 (18) | 75 (21) | <0.001[b] |
| Most common rheumatic disease diagnoses[$] | | | | |
| Inflammatory arthritis* | 61 (41.9) | 45 (75.0) | 15 (25.0) | <0.001 |
| Gout | 21 (20.0) | 0 (0) | 21 (100.0) | <0.001 |
| Connective Tissue Disease and Other* | 25 (23.8) | 11 (45.8) | 13 (54.2) | 0.338 |
| | | | | |
| Most common symptoms | | | | |
| Asymptomatic | 4 (4.6) | 1 (25.0) | 3 (75.0) | 0.621[a] |
| Fever | 67 (63.8) | 37 (56.1) | 29 (43.0) | 0.645 |
| Cough | 76 (72.4) | 44 (58.7) | 31 (41.3) | 0.152 |
| Shortness of breath | 57 (54.3) | 26 (47.3) | 29 (52.7) | 0.122 |
| Myalgia | 35 (33.3) | 24 (70.6) | 10 (29.4) | 0.020 |
| Number of symptoms (Median, IQR) | 4 (2) | 4 (3) | 4 (3) | 0.0341[b] |
| No comorbidities | 38 (36.2) | 32 (84.2) | 6 (15.8) | <0.001 |
| Most common comorbidities | | | | |
| Cancer | 4 (3.8) | 0 (0.0) | 4 (100.0) | 0.040[a] |
| Cerebrovascular disease | 7 (6.7) | 0 (0.0) | 7 (100.0) | 0.003[a] |
| COPD / asthma | 13 (12.4) | 3 (23.1) | 10 (76.9) | 0.015 |
| CVD | 26 (24.8) | 8 (32.0) | 17 (68.0) | 0.010 |
| Diabetes | 11 (10.5) | 0 (0.0) | 11 (100.0) | <0.001 |
| Hypertension | 32 (30.5) | 10 (32.3) | 21 (67.7) | 0.003 |
| Interstitial lung disease | 3 (2.9) | 0 (0.0) | 3 (100.0) | 0.092[a] |
| Neurological/ Neuromuscular disease | 3 (2.9) | 0 (0.0) | 3 (100.0) | 0.092[a] |
| Obesity | 6 (5.7) | 4 (66.7) | 2 (33.3) | 0.686[a] |
| Renal disease | 10 (9.5) | 0 (0.0) | 10 (100.0) | <0.001 |
| Number of comorbidities (Median, IQR) | 1 (2) | 0 (1) | 2 (2) | <0.001[b] |
| Smoking Status | | | | |
| Never | 62 (59.6) | 37 (59.7) | 25 (40.3) | 0.069 |
| Ever | 23 (22.1) | 7 (31.8) | 15 (68.2) | |
| Unknown | 19 (18.3) | | | |
| Medication prior to COVID-19 diagnosis | | | | |
| Glucocorticoids | 15 (14.3) | 2 (14.3) | 12 (85.7) | 0.001 |
| Glucocorticoid-equivalent >/= 10mg or more | 7 (6.7) | 0 (0.0) | 6 (100.0) | 0.008[a] |
| csDMARD monotherapy | 33 (31.4) | 22 (68.8) | 10 (31.2) | 0.049 |
| b/tsDMARD (monotherapy or in combination with csDMARD) | 37 (35.2) | 28 (75.7) | 9 (24.3) | 0.001 |

| | | | | |
|---|---|---|---|---|
| No complications | 19 (18.1) | 55 (64.7) | 30 (35.3) | <0.001 |
| Deceased | 10 (9.5) | 0 (0.0) | 10 (100.0) | <0.001[a] |

N (column %) for categorical variables, unless otherwise specified
Column numbers and percentages many not sum to 100 due to missing values$ Patients could be diagnosed with more than one rheumatic diseases
∞ P-value from Pearson's Chi Square test, unless a=Fisher's Exact test and b=Mann Whitney U test
* The inflammatory arthritis group was composed of rheumatoid arthritis (37), psoriatic arthritis (13), axial spondyloarthritis (8), juvenile idiopathic arthritis (2), other inflammatory arthritis (2). The connective tissue disease and others group was comprised of systemic lupus erythematosus (5), polymyalgia rheumatica (4), Sjogrens syndrome (4), giant cell arteritis (4), and others (11).
[&]Symptoms with frequency >30% reported

**Table 2. Association between demographic and clinical characteristics and COVID-19 hospitalisation status.**

| | | All significant variable model | | Most parsimonious model | |
|---|---|---|---|---|---|
| | Unadjusted OR (95% CI) | Adjusted OR (95%CI)* | Adjusted p-value* | Adjusted OR (95%CI)[&] | Adjusted p-value[&] |
| | | | | | |
| Female | 0.45 (0.20-1.02) | 0.33 (0.05-2.23) | | 0.34 (0.09-1.36) | 0.128 |
| Age (years) | 1.08 (1.05-1.11) | 1.04 (0.97-1.10) | 0.224 | 1.06 (1.01-1.10) | 0.010 |
| Most common rheumatic disease diagnoses[$] | | | | | |
| Inflammatory arthritis | 0.11 (0.05-0.28) | 0.14 (0.02-0.95) | 0.044 | 0.09 (0.02-0.32) | <0.001 |
| Gout | — | | | | |
| Connective Tissue Disease and Other | 1.56 (0.62 - 3.92) | | | | |
| Asymptomatic | 0.38 (0.04-3.77) | | | | |
| Most common symptoms | | | | | |
| Fever | 0.83 (0.37-1.85) | | | | |
| Headache | 1.16 (0.05-0.46) | 0.25 (0.02-3.58) | 0.310 | | |
| Sore throat | 0.06 (0.01-0.29) | 0.30 (0.03-2.79) | 0.292 | | |
| Cough | 0.53 (0.22-1.27) | | | | |
| Shortness of breath | 1.86 (0.84-4.09) | | | | |
| Arthralgia | 0.23 (0.05-1.13) | | | | |
| Myalgia | 0.36 (0.15 -0.87) | 0.92 (0.15-5.81) | 0.931 | | |
| Chest pain | 0.25 (0.07-0.95) | 0.91 (0.08-9.92) | 0.941 | | |
| Abdominal pain | 3.21 (0.59-17.4) | | | | |
| Diarrhoea / Vomiting / Nausea | 3.24 (1.28-8.17) | 1.81 (0.26-12.68) | 0.551 | | |
| Rhinorrhoea | 0.18 (0.02-1.56) | | | | |
| Irritation / Confusion | 3.75 (0.38-37.3) | | | | |
| Malaise | 1.72 (0.71-4.16) | | | | |
| Anosmia | 0.08 (0.01-0.64) | 0.60 (0.03-11.49) | 0.737 | | |
| Dysgeusia | 0.13 (0.02-1.08) | | | | |
| Fatigue | 0.49 (0.20-1.18) | | | | |
| Number of symptoms (Median, IQR) | 0.79 (0.66-0.95) | 1.08 (0.59-1.99) | 0.798 | | |
| No comorbidities | 0.11 (0.04-0.30) | 0.76 (0.09-6.58) | 0.802 | | |
| Most common comorbidities | | | | | |
| Cancer | — | | | | |
| Cerebrovascular disease | — | | | | |

| | | | | | |
|---|---|---|---|---|---|
| COPD / asthma | 4.77 (1.23-18.54) | 3.09 (0.16-60.07) | 0.456 | | |
| CVD | 3.40 (1.31-8.85) | 0.11 (0.01-1.88) | 0.129 | | |
| Diabetes | — | | | | |
| Hypertension | 3.71 (1.52-9.08) | 0.56 (0.04-7.94) | 0.668 | | |
| Interstitial lung disease | — | | | | |
| Neurological / Neuromuscular disease | — | | | | |
| Obesity | 0.58 (0.10-3.30) | | | | |
| Psychiatric condition | — | | | | |
| Renal disease | — | | | | |
| Number of comorbidities (Median, IQR) | 3.01 (1.92-4.72) | 2.99 (0.59-15.02) | 0.184 | 1.93 (1.11-3.35) | 0.020 |
| Smoking Status | | | | | |
| Never | ref. | | 0.889 | | |
| Ever | 3.17 (1.18-8.89) | 1.19 (0.10-13.68) | | | |
| Medication prior to COVID-19 diagnosis | | | | | |
| Glucocorticoids | 9.26 (1.95-43.89) | 18.14 (1.13-290.81) | 0.041 | 15.01 (1.77-127.16) | 0.013 |
| Glucocorticoid-equivalent >/= 10mg or more | — | | | | |
| csDMARD monotherapy | 0.42 (0.17-1.00) | | | | |
| b/tsDMARD (monotherapy or in combination with csDMARD) | 0.24 (0.10-0.58) | 1.36 (0.19-9.72) | 0.557 | | |

*Multivariable model including all variables with significant effect estimates in the univariable logistic regression analyses

[&] Most parsimonious multivariable model; including age, gender and only those variables retaining statistical significance

## Supplementary Material

rkab031_Supplementary_Data

