# Predictors of hospitalization in patients with rheumatic disease and COVID-19 in Ireland: data from the COVID-19 global rheumatology alliance registry

**DOI:** 10.1093/rap/rkab031

**Published:** 2021-05-13

**Authors:** Richard Conway, Elena Nikiphorou, Christiana A Demetriou, Candice Low, Kelly Leamy, John G Ryan, Ronan Kavanagh, Alexander D Fraser, John J Carey, Paul O’Connell, Rachael M Flood, Ronan H Mullan, David J Kane, Philip C Robinson, Jean W Liew, Rebecca Grainger, Geraldine M McCarthy, for the COVID-19 Global Rheumatology Alliance

**Affiliations:** Department of Rheumatology, St James’s Hospital; Trinity College Dublin, Dublin, Ireland; Department of Rheumatology; Centre for Rheumatic Diseases, King’s College London, London, UK; Department of Primary Care and Population Health, University of Nicosia Medical School, Nicosia, Cyprus; Department of Rheumatology, St. Vincent’s University Hospital, Elm Park; Department of Rheumatology, Mater Misericordiae Hospital, Dublin; Department of Rheumatology, Cork University Hospital, Cork; Galway Clinic, Galway; Department of Rheumatology, University Hospitals Limerick; Graduate Entry Medical School, University of Limerick, Limerick; Department of Rheumatology, Galway University Hospitals; Medicine, National University of Ireland Galway, Galway; Department of Rheumatology, Beaumont Hospital; Royal College of Surgeons in Ireland; Trinity College Dublin, Dublin, Ireland; Department of Rheumatology, Tallaght University Hospital, Dublin, Ireland; Trinity College Dublin, Dublin, Ireland; Department of Rheumatology, Tallaght University Hospital, Dublin, Ireland; Trinity College Dublin, Dublin, Ireland; Department of Rheumatology, Tallaght University Hospital, Dublin, Ireland; University of Queensland Faculty of Medicine, Brisbane, Queensland, Australia; Section of Rheumatology, Boston University School of Medicine, Boston, MA, USA; Department of Medicine, University of Otago, Wellington, New Zealand; Department of Rheumatology, Mater Misericordiae Hospital, Dublin

**Keywords:** rheumatic disease, COVID-19, biologics, hospitalization

## Abstract

**Objectives:**

Given the limited data regarding the risk of hospitalization in patients with rheumatic disease and coronavirus disease 2019 (COVID-19) in Ireland, we used the COVID-19 Global Rheumatology Alliance (GRA) registry data to study outcomes and their predictors. The primary objective was to explore potential predictors of hospitalization.

**Methods:**

We examined data on patients and their disease-related characteristics entered in the COVID-19 GRA provider registry from Ireland (from 24 March 2020 to 31 August 2020). Multivariable logistic regression was used to assess the association of demographic and clinical characteristics with hospitalization.

**Results:**

Of 105 patients, 47 (45.6%) were hospitalized and 10 (9.5%) died. Multivariable logistic regression analysis showed that age [odds ratio (OR) = 1.06, 95% CI 1.01, 1.10], number of co-morbidities (OR = 1.93, 95% CI 1.11, 3.35) and glucocorticoid use (OR = 15.01, 95% CI 1.77, 127.16) were significantly associated with hospitalization. A diagnosis of inflammatory arthritis was associated with lower odds of hospitalization (OR = 0.09, 95% CI 0.02, 0.32).

**Conclusion:**

Increasing age, co-morbidity burden and glucocorticoid use were associated with hospitalization, whereas a diagnosis of inflammatory arthritis was associated with lower odds of hospitalization.

Key messages

- Increasing age, co-morbidity burden and glucocorticoid use are associated with higher odds of hospitalization.
- A diagnosis of inflammatory arthritis is associated with lower odds of hospitalization.
- Insights from this study can help direct local strategies for the management of coronavirus disease 2019 in this patient group.

## Introduction

Infection with the severe acute respiratory syndrome coronavirus 2 (SARS-CoV-2) and the subsequent coronavirus disease 2019 (COVID-19) has caused considerable morbidity and mortality worldwide. Patients with rheumatic diseases are generally at increased risk of infection owing to both their disease with the associated co-morbidities and their immunomodulatory or suppressive treatments [1]. Whether this applies to SARS-CoV-2 infection and the magnitude of any such risk remains unclear.

The COVID-19 Global Rheumatology Alliance (C19-GRA) aims to address the knowledge gap about COVID-19 for patients with rheumatic disease [2]. The C19-GRA has reported data on the first 600 cases in its physician-reported case registry [3]. In these patients, glucocorticoid doses of prednisolone equivalent ≥10 mg/day were associated with increased risk of hospitalization, whereas TNF-α inhibitors were associated with a decreased risk of hospitalization [3]. Overall, as with the general population, the greatest risk of negative outcomes was associated with increasing age and co-morbidities.

Geographical variations in COVID-19 outcomes have been reported [4–8]. Our aim was to report the outcomes and their determinants in patients in Ireland with rheumatic disease who developed COVID-19.

## Methods

### COVID-19 Global Rheumatology Alliance

Data regarding individuals with rheumatic disease who develop COVID-19 are entered into one of two parallel international data portals hosted in the USA and the UK. Details of the C19-GRA registries have been published previously [2, 3, 9].

### Data collection

Patients in this study were entered into the C19-GRA provider registry from 24 March 2020 to 31 August 2020.

Data collected on baseline rheumatic disease status, including demographic and clinical variables, were as follows: age, sex, smoking status, rheumatic disease diagnosis, disease activity and co-morbidities. Medications were categorized as previously described (Supplementary Data S1, available at *Rheumatology Advances in Practice* online) [3]. Rheumatic diseases were categorized as inflammatory arthritis, gout, vasculitis, CTDs and others (Supplementary Data S2, available at *Rheumatology Advances in Practice* online). Data collected regarding COVID-19 infection included the method of diagnosis, place of diagnosis, COVID-19 symptoms and the outcomes of COVID-19 disease, including hospitalization, ventilation and death.

Continuous variables were reported as the median [interquartile range (IQR)]. Categorical variables were reported as the number and percentage. In univariable analyses, differences according to hospitalization status and mortality were compared using χ^2^ tests or Fisher’s exact tests, as appropriate, for categorical variables and Mann–Whitney *U*-tests for continuous variables. The independent associations between demographic and disease-specific features with the odds of COVID-19 hospitalization were estimated using univariable followed by multivariable-adjusted logistic regression and reported as odds ratios (ORs) and 95% CIs. We constructed two multivariable models. The first model included all variables with significant effect sizes (*P* < 0.05) in the univariable logistic regression analyses, whereas the second model was the most parsimonious model including age, biologic sex and those variables retaining statistical significance. We did not proceed to logistic regression analyses with mortality as the outcome owing to the small number of deaths.

This study was approved by the Irish National Research Ethics Committee for COVID-19 (20-NREC-COV-010). The committee waived the need for written informed patient consent because the data were fully anonymized.

## Results

The characteristics of the 105 included patients are shown in Table 1. The median age was 59 years, and 61% were female. The most common rheumatic disease grouping was inflammatory arthritis (61 of 105, 41.9%) followed by CTD and others (25 of 105, 23.8%) and gout (21 of 105, 20%). Co-morbidities were present in almost two-thirds of patients (67 of 105, 63.8%); the two most common were hypertension (32 of 105, 30.5%) and cardiovascular disease (26 of 105, 24.8%). Before COVID-19 diagnosis, glucocorticoids were prescribed to 15 of 105 (14.3%), conventional synthetic DMARD (csDMARD) monotherapy to 33 of 105 (31.4%) and biological DMARDs (bDMARDs) or targeted synthetic DMARDs (tsDMARDs) to 37 of 105 (35.2%). Almost half of reported cases were hospitalized (47 of 105, 45.6%), and 10 (9.5%) died.

**Table 1:** Characteristics of patients with rheumatic disease diagnosed with coronavirus disease 2019, for all participants and by hospitalization status

Characteristic	All participants (*n* = 105)	Not hospitalized (*n* = 56)	Hospitalized (*n* = 47)	*P*-value*
	***n* (%)**	***n* (%)**	***n* (%)**	
Biologic sex				
Female	64 (61.0)	39 (61.9)	24 (38.10)	0.054
Male	41 (39.0)	17 (42.5)	23 (57.5)	
Age, years				
18–29	3 (2.9)	2 (66.7)	1 (33.3)	<0.001^†^
30–49	25 (23.8)	21 (84.0)	4 (16.0)	
50–65	32 (30.5)	22 (71.0)	9 (29.0)	
>65	45 (42.9)	11 (25.0)	33 (75.0)	
Median (IQR)	59 (27)	51 (18)	75 (21)	<0.001^‡^
Most common rheumatic disease diagnoses^a^				
Inflammatory arthritis^b^	61 (41.9)	45 (75.0)	15 (25.0)	<0.001
Gout	21 (20.0)	0 0	21 (100.0)	<0.001
CTD and other^b^	25 (23.8)	11 (45.8)	13 (54.2)	0.338
Most common symptoms^c^				
Asymptomatic	4 (4.6)	1 (25.0)	3 (75.0)	0.621^†^
Fever	67 (63.8)	37 (56.1)	29 (43.0)	0.645
Cough	76 (72.4)	44 (58.7)	31 (41.3)	0.152
Shortness of breath	57 (54.3)	26 (47.3)	29 (52.7)	0.122
Myalgia	35 (33.3)	24 (70.6)	10 (29.4)	0.020
Number of symptoms, median (IQR)	4 (2)	4 (3)	4 (3)	0.0341^‡^
No co-morbidities	38 (36.2)	32 (84.2)	6 (15.8)	<0.001
Most common co-morbidities				
Cancer	4 (3.8)	0 (0.0)	4 (100.0)	0.040^†^
Cerebrovascular disease	7 (6.7)	0 (0.0)	7 (100.0)	0.003^†^
COPD/asthma	13 (12.4)	3 (23.1)	10 (76.9)	0.015
Cardiovascular disease	26 (24.8)	8 (32.0)	17 (68.0)	0.010
Diabetes	11 (10.5)	0 (0.0)	11 (100.0)	<0.001
Hypertension	32 (30.5)	10 (32.3)	21 (67.7)	0.003
Interstitial lung disease	3 (2.9)	0 (0.0)	3 (100.0)	0.092^†^
Neurological/neuromuscular disease	3 (2.9)	0 (0.0)	3 (100.0)	0.092^†^
Obesity	6 (5.7)	4 (66.7)	2 (33.3)	0.686^†^
Renal disease	10 (9.5)	0 (0.0)	10 (100.0)	<0.001
Number of co-morbidities, median (IQR)	1 (2)	0 (1)	2 (2)	<0.001^‡^
Smoking status				
Never	62 (59.6)	37 (59.7)	25 (40.3)	0.069
Ever	23 (22.1)	7 (31.8)	15 (68.2)	
Unknown	19 (18.3)			
Medication before COVID-19 diagnosis				
Glucocorticoids	15 (14.3)	2 (14.3)	12 (85.7)	0.001
Glucocorticoid equivalent ≥10 mg	7 (6.7)	0 (0.0)	6 (100.0)	0.008^†^
csDMARD monotherapy	33 (31.4)	22 (68.8)	10 (31.2)	0.049
b/tsDMARD (monotherapy or in combination with csDMARD)	37 (35.2)	28 (75.7)	9 (24.3)	0.001
No complications	19 (18.1)	55 (64.7)	30 (35.3)	<0.001
Deceased	10 (9.5)	0 (0.0)	10 (100.0)	<0.001^†^

Values are *n* (column %) for categorical variables, unless otherwise specified. Column numbers and percentages may not sum to 100 owing to missing values.

a Patients could be diagnosed with more than one rheumatic disease.

b The inflammatory arthritis group was composed of RA (37), PsA (13), axial spondyloarthritis (8), JIA (2) and other inflammatory arthritis (2). The CTD and others group was composed of SLE (5), PMR (4), SS (4), GCA (4) and others (11).

c Symptoms with frequency >30% are reported.

* *P*-value from Pearson’s χ^2^ test.

† *P*-value from Fisher’s exact test.

‡ *P*-value from Mann–Whitney *U*-test.

bDMARD: biological DMARD; COPD: chronic obstructive pulmonary disease; COVID-19: coronavirus disease 2019; csDMARD: conventional synthetic DMARD; IQR: interquartile range; tsDMARD: targeted synthetic DMARD.

When compared with patients not hospitalized, hospitalized patients were older, with a median age of 75 years compared with 51 years (*P* < 0.001; Table 2). A higher proportion of patients with gout were hospitalized (100%, *P* < 0.001), whereas a lower proportion of patients with inflammatory arthritis were hospitalized (25%, *P* < 0.001). Patients with no co-morbidities had a lower frequency of hospitalization (6 of 47, 15.8%, *P* < 0.001), and hospitalized patients had a higher median number of co-morbidities (median 2 *vs* 0, *P* < 0.001). Hospitalization was more common in patients on glucocorticoids (85.7%, *P* = 0.001), with patients on ≥10 mg prednisolone equivalent all hospitalized (100%, *P* = 0.008). Hospitalization was less frequent among those prescribed csDMARD monotherapy (31.2%, *P* = 0.049) and those prescribed bDMARDs or tsDMARDs either alone or in combination with csDMARDs (24.3%, *P* = 0.001).

**Table 2:** Association between demographic and clinical characteristics and coronavirus disease 2019 hospitalization status

Characteristic		All significant variable model		Most parsimonious model	
	Unadjusted OR (95% CI)	Adjusted OR (95% CI)**^a^**	Adjusted *P*-value**^a^**	Adjusted OR (95% CI)**^b^**	Adjusted *P*-value**^b^**
Female	0.45 (0.20, 1.02)	0.33 (0.05, 2.23)	–	0.34 (0.09, 1.36)	0.128
Age, years	1.08 (1.05, 1.11)	1.04 (0.97, 1.10)	0.224	1.06 (1.01, 1.10)	0.010
Most common rheumatic disease diagnoses	–	–	–	–	–
Inflammatory arthritis	0.11 (0.05, 0.28)	0.14 (0.02, 0.95)	0.044	0.09 (0.02, 0.32)	<0.001
Gout	–	–	–	–	–
CTD and other	1.56 (0.62, 3.92)	–	–	–	–
Asymptomatic	0.38 (0.04, 3.77)	–	–	–	–
Most common symptoms	–	–	–	–	–
Fever	0.83 (0.37, 1.85)	–	–	–	–
Headache	1.16 (0.05, 0.46)	0.25 (0.02, 3.58)	0.310	–	–
Sore throat	0.06 (0.01, 0.29)	0.30 (0.03, 2.79)	0.292	–	–
Cough	0.53 (0.22, 1.27)	–	–	–	–
Shortness of breath	1.86 (0.84, 4.09)	–	–	–	–
Arthralgia	0.23 (0.05, 1.13)	–	–	–	–
Myalgia	0.36 (0.15, 0.87)	0.92 (0.15, 5.81)	0.931	–	–
Chest pain	0.25 (0.07, 0.95)	0.91 (0.08, 9.92)	0.941	–	–
Abdominal pain	3.21 (0.59, 17.4)	–	–	–	–
Diarrhoea/vomiting/nausea	3.24 (1.28, 8.17)	1.81 (0.26, 12.68)	0.551	–	–
Rhinorrhoea	0.18 (0.02, 1.56)	–	–	–	–
Irritation/confusion	3.75 (0.38, 37.3)	–	–	–	–
Malaise	1.72 (0.71, 4.16)	–	–	–	–
Anosmia	0.08 (0.01, 0.64)	0.60 (0.03, 11.49)	0.737	–	–
Dysgeusia	0.13 (0.02, 1.08)	–	–	–	–
Fatigue	0.49 (0.20, 1.18)	–	–	–	–
Number of symptoms, median (IQR)	0.79 (0.66, 0.95)	1.08 (0.59, 1.99)	0.798	–	–
No co-morbidities	0.11 (0.04, 0.30)	0.76 (0.09, 6.58)	0.802	–	–
Most common co-morbidities	–	–	–	–	–
Cancer	–	–	–	–	–
Cerebrovascular disease	–	–	–	–	–
COPD/asthma	4.77 (1.23, 18.54)	3.09 (0.16, 60.07)	0.456	–	–
Cardiovascular disease	3.40 (1.31, 8.85)	0.11 (0.01, 1.88)	0.129	–	–
Diabetes	–	–	–	–	–
Hypertension	3.71 (1.52, 9.08)	0.56 (0.04, 7.94)	0.668	–	–
Interstitial lung disease	–	–	–	–	–
Neurological/neuromuscular disease	–	–	–	–	–
Obesity	0.58 (0.10, 3.30)	–	–	–	–
Psychiatric condition	–	–	–	–	–
Renal disease	–	–	–	–	–
Number of co-morbidities, median (IQR)	3.01 (1.92, 4.72)	2.99 (0.59, 15.02)	0.184	1.93 (1.11, 3.35)	0.020
Smoking status	–	–	–	–	–
Never	Reference	–	0.889	–	–
Ever	3.17 (1.18, 8.89)	1.19 (0.10, 13.68)	–	–	–
Medication before COVID-19 diagnosis	–	–	–	–	–
Glucocorticoids	9.26 (1.95, 43.89)	18.14 (1.13, 290.81)	0.041	15.01 (1.77, 127.16)	0.013
Glucocorticoid equivalent ≥10 mg	–	–	–	–	–
csDMARD monotherapy	0.42 (0.17, 1.00)	–	–	–	–
b/tsDMARD (monotherapy or in combination with csDMARD)	0.24 (0.10, 0.58)	1.36 (0.19, 9.72)	0.557	–	–

a Multivariable model, including all variables with significant effect estimates in the univariable logistic regression analyses.

b Most parsimonious multivariable model, including age, biologic sex and only those variables retaining statistical significance.

bDMARD: biological DMARD; COPD: chronic obstructive pulmonary disease; COVID-19: coronavirus disease 2019; csDMARD: conventional synthetic DMARD; IQR: interquartile range; OR: odds ratio; tsDMARD: targeted synthetic DMARD.

In the multivariable logistic regression model, including all variables with significant effects estimates in univariable logistic regression analyses, glucocorticoid use was associated with higher odds of hospitalization (OR = 18.14, 95% CI 1.13, 290.81, *P* = 0.041; Table 2). A diagnosis of inflammatory arthritis was associated with lower odds of hospitalization (OR = 0.18, 95% CI 0.02, 0.95, *P* = 0.044). In the most parsimonious multivariable logistic regression model, age (OR = 1.06, 95% CI 1.01, 1.10, *P* = 0.01), number of co-morbidities (OR = 1.93, 95% CI 1.11, 3.35, *P* = 0.02) and glucocorticoid use (OR = 15.01, 95% CI 1.77, 127.16, *P* = 0.013) were associated with increased odds of hospitalization. A diagnosis of inflammatory arthritis was associated with lower odds of hospitalization (OR = 0.09, 95% CI 0.02, 0.32, *P* < 0.001). csDMARDs and b/tsDMARDs were not associated with hospitalization in the multivariable analysis.

In unadjusted analyses, deceased patients were older (median 80.5 *vs* 58 years; *P* = 0.001). No patients with no co-morbidities died of COVID-19 (0%, *P* = 0.013), and patients who died had a median of 3.5 co-morbidities (median 3.5 *vs* 0, *P* < 0.001; Supplementary Table S1, available at *Rheumatology Advances in Practice* online). Specific co-morbidities associated with mortality included cardiovascular disease (23.1%, *P* = 0.014), diabetes mellitus (36.4%, *P* = 0.01), hypertension (21.9%, *P* = 0.008), renal disease (40%, *P* = 0.007) and interstitial lung disease (66.7%, *P* = 0.023).

## Discussion

This is the largest report of people with rheumatic disease and COVID-19 from Ireland. We identified factors associated with higher odds of hospitalization, including increasing age, number of co-morbidities, a diagnosis of gout and baseline glucocorticoid use. A diagnosis of inflammatory arthritis showed a protective association with hospitalization.

Our findings of the association of increasing age and glucocorticoid use with hospitalization are consistent with the findings from C19-GRA [3]. This highlights that glucocorticoid use should be limited in favour of longer-term disease-modifying therapies. The C19-GRA reported bDMARDs as being associated with a reduced risk of hospitalization, which has some biologic plausibility owing to the potential for reduction of the hyperinflammatory state seen in severe COVID-19 [3, 10, 11]. Several bDMARDs have been proposed as treatment for severe COVID-19, but results of many controlled trials have been disappointing [12, 13]. The RECOVERY and REMAP-CAP trials have demonstrated evidence of modest benefit from the use of the IL-6 receptor antagonists tocilizumab and sarilumab in COVID-19 [14, 15]. In our population, the apparent benefit of both csDMARDs and bDMARDs in the univariable analysis disappeared after controlling for the underlying diagnostic group. These agents, and particularly bDMARDs, are more likely to be used in patients with a diagnosis of inflammatory arthritis, and the reduced risk of hospitalization with these agents might represent confounding by indication. The results of ongoing randomized controlled trials of other bDMARDs in COVID-19 will provide clarity.

Our study has several limitations. Although the low number of cases of people with rheumatic diseases and COVID-19 in Ireland entered in the C-19 GRA registry limits the power of our study, this study explores associations between patient and disease characteristics and outcomes in Ireland. Potential overfitting of multivariable models can occur with small sample sizes and is one reason we did not proceed to multivariable analysis for mortality. Once further cases are entered into the GRA registries, we will be able to conduct more detailed analyses in the future. As a physician-entered registry, the C19-GRA is limited by selection bias, with more severe cases likely to be entered, which is likely to explain why 100% of gout cases were hospitalized. Given that there is no denominator population for these data, no inferences can be drawn about the incidence of COVID-19 in patients with rheumatic diseases. The C19-GRA is restricted to patients who have both rheumatic diseases and COVID-19; therefore, no comparisons can be made between this group and the general rheumatic disease population or with patients without rheumatic disease who develop COVID-19. Our finding of gout as a risk factor for hospitalization, although highly significant because all gout cases were hospitalized, might reflect selection bias and requires future evaluation. Gout is usually managed in primary care in Ireland, and given that the C19-GRA is a rheumatologist-entered registry these cases could be less likely to be entered. Patients with rheumatic diseases might have behaved differently with regard to risk behaviour during COVID-19. In Ireland, a strict national lockdown was in place for a large part of the study period (including when most cases were reported). Individuals judged to be at high risk at the start of the pandemic, including many rheumatic diseases, were specifically advised to stay at home as much as possible and to avoid social interactions [16].

In conclusion, our findings confirm that increasing age, co-morbidity burden and glucocorticoid use are associated with hospitalization with COVID-19 in patients with rheumatic diseases. An underlying diagnosis of inflammatory arthritis appears to be associated with lower risk of hospitalization. The previously reported beneficial effects of bDMARDs might be mediated by underlying diagnosis rather than a specific effect.

## Supplementary Material

rkab031_Supplementary_Data

## Data Availability

Request for access to data from the registry should be made to the Data Access and Sharing Committee of the COVID-19 Global Rheumatology Alliance. The data underlying this article are available on reasonable request to the corresponding author.
