# Supplementary material for: Predictors of hospitalization in patients with rheumatic disease and COVID-19 in Ireland: data from the COVID-19 global rheumatology alliance registry
Source: Rheumatol Adv Pract. 2021 May 13;5(2):rkab031. doi: 10.1093/rap/rkab031 (PMC8244588; doi:10.1093/rap/rkab031)
Supplement: rkab031_Supplementary_Data [file rkab031_supplementary_data.zip › 21-039 Supplementary Material.docx]

**Supplementary Data S1. Medication categorisation**

Medications prior to COVID-19 were categorised as glucocorticoids (further categorised as prednisolone equivalent <10mg/day or ≥10mg/day), conventional synthetic disease-modifying antirheumatic drugs (csDMARDs; antimalarials (including hydroxychloroquine, chloroquine, mepacrine/quinacrine), apremilast, thiopurines (azathioprine, 6-mercaptopurine), cyclosporine, leflunomide, methotrexate, mycophenolate mofetil/mycophenolic acid, sulfasalazine, tacrolimus, thalidomide/lenalidomide), biologic and targeted synthetic DMARD (bDMARD/tsDMARD; abatacept, belimumab, CD-20 inhibitors (including rituximab, ofatumumab), cyclophosphamide, IL-1 inhibitors (including anakinra, canakinumab, rilonacept), IL-6 inhibitors (including tocilizumab, sarilumab), IL-12/23 inhibitors (ustekinumab), IL-23 inhibitors (guselkumab, risankizumab), IL-17 inhibitors (including secukinumab, ixekizumab), janus kinase (JAK) inhibitors (including tofacitinib, baricitinib, upadacitinib), TNF-inhibitors (including infliximab, etanercept, adalimumab, golimumab, certolizumab, and their biosimilars)).

**Supplementary Data S2. Rheumatic disease categorisation**

Rheumatic diseases were categorised as 1) inflammatory arthritis (axial spondyloarthritis (including ankylosing spondylitis), psoriatic arthritis, other spondyloarthritis (including reactive arthritis), juvenile idiopathic arthritis (JIA (including systemic JIA), rheumatoid arthritis, other inflammatory arthritis), 2) gout, 3) vasculitis, connective tissue diseases, and others (ANCA-associated vasculitis , other vasculitis including Kawasaki disease, anti-phospholipid antibody syndrome, autoinflammatory syndrome (including TRAPS, CAPS, FMF), Behcet's disease, chronic recurrent multifocal osteomyelitis, giant cell arteritis, IgG4-related disease, inflammatory myopathy (e.g. dermatomyositis, polymyositis), inclusion body myositis (IBM), mixed connective tissue disease, ocular inflammation, polymyalgia rheumatica, sarcoidosis, Sjogren's syndrome, systemic lupus erythematosus, systemic sclerosis, undifferentiated connective tissue disease, localised scleroderma (morphea)).

**Supplementary Table S1. Demographic and clinical factors of patients with rheumatic disease diagnosed with COVID-19 by deceased status**

|  | **Not deceased (n=95)** | **Deceased (n=10)** |  |
| --- | --- | --- | --- |
|  | **N(%)*** | **N(%)*** | **p-value**^∞^ |
| **Gender** |  |  |  |
| Female | 58 (90.6) | 6 (9.4) | 1.000 |
| Male | 37 (90.2) | 4 (9.8) |  |
| **Age (years)** |  |  |  |
| 18-29 | 3 (100.0) | 0 (0.0) | 0.096 |
| 30-49 | 25 (100.0) | 0 (0.0) |  |
| 50-65 | 30 (93.8) | 2 (6.2) |  |
| >65 | 37 (82.2) | 8 (17.8) |  |
| Median (IQR) | 58 (26) | 80.5 (17) | 0.001^a^ |
| **Most common rheumatic disease diagnoses^$^** |  |  |  |
| Inflammatory arthritis | 57 (93.4) | 4 (6.6) | 0.314 |
| Gout | 17 (81.0) | 4 (19.0) | 0.110 |
| Connective Tissue Disease and Other | 23 (92.0) | 2 (8.0) | 1.000 |
| **Asymptomatic** | 3 (75) | 1 (25) | 0.392 |
| **Most common symptoms** |  |  |  |
| Fever | 62 (92.5) | 5 (7.5) | 0.490 |
| Headache | 29 (100.0) | 0 (0.0) | 0.058 |
| Sore throat | 25 (100.0) | 0 (0.0) | 0.113 |
| Cough | 70 (92.1) | 6 (7.9) | 0.458 |
| Shortness of breath | 51 (89.5) | 6 (10.5) | 0.751 |
| Arthralgia | 10 (90.9) | 1 (9.1) | 1.000 |
| Myalgia | 32 (91.4) | 3 (8.6) | 1.000 |
| Chest pain | 15 (100.0) | 0 (0.0) | 0.351 |
| Abdominal pain | 5 (71.4) | 2 (28.6) | 0.133 |
| Diarrhoea/Vomiting/Nausea | 22 (81.5) | 5 (18.5) | 0.120 |
| Rhinorrhoea | 8 (100.0) | 0 (0.0) | 1.000 |
| Irritation/Confusion | 1 (25.0) | 3 (75.0) | 0.002 |
| Malaise | 24 (85.7) | 4 (14.3) | 0.451 |
| Anosmia | 13 (100.0) | 0 (0.0) | 0.357 |
| Dysgeusia | 9 (100.0) | 0 (0.0) | 0.357 |
| Fatigue | 29 (90.5) | 10 (9.5) | 0.275 |
| **Number of symptoms (Median, IQR)** | 4 (2) | 4.5 (2) | 0.753 ^a^ |
| **No comorbidities** | 38 (100.0) | 0 (0.0) | 0.013 |
| **Most common comorbidities** |  |  |  |
| Cancer | 4 (100.0) | 0 (0.0) | 1.000 |
| Cerebrovascular disease | 5 (71.4) | 2 (28.6) | 0.133 |
| COPD / asthma | 11 (84.6) | 2 (15.4) | 0.609 |
| CVD | 20 (76.9) | 6 (23.1) | 0.014 |
| Diabetes | 7 (63.6) | 4(36.4) | 0.010 |
| Hypertension | 25.0 (78.1) | 7 (21.9) | 0.008 |
| Interstitial lung disease | 1 (33.3) | 2 (66.7) | 0.023 |
| Neurological / Neuromuscular disease | 2 (66.7) | 1 (33.3) | 0.262 |
| Obesity | 4 (66.7) | 2 (33.3) | 0.100 |
| Psychiatric condition | 2 (66.7) | 1 (33.3) | 0.262 |
| Renal disease | 6 (60.0) | 4 (40.0) | 0.007 |
| **Number of comorbidities (Median, IQR)** | 0 (2) | 3.5 (2) | <0.001 ^a^ |
| **Smoking Status** |  |  |  |
| Never | 55 (88.7) | 7 (11.3) | 1.000 |
| Ever | 21 (91.3) | 2 (8.7) |  |
| **Medication prior to COVID-19 diagnosis** |  |  |  |
| Steroids | 13 (86.7) | 2 (13.3) | 0.633 |
| Steroids 10mg or more | 6 (85.7) | 1 (14.3) | 0.515 |
| csDMARD monotherapy | 29 (87.9) | 4 (12.1) | 0.721 |
| b/tsDMARD (monotherapy or in combination with csDMARD) | 36 (97.3) | 1 (2.7) | 0.095 |
| **No complications** | 80 (93.0) | 6 (7.0) | 0.079 |
| **Most common complications** |  |  |  |
| ARDS | 2 (33.3) | 4 (66.7) | 0.001 |
| Sepsis | 3 (60.0) | 2 (40.0) | 0.070 |
| Concomitant Infection | 5 (100.0) | 0 (0.0) | 1.000 |
| Thromboembolism | 1 (100.0) | 0 (0.0) | 1.000 |
| AKI or renal failure | 2 (66.7) | 1 (33.3) | 0.262 |

*N (row %) for categorical variables

^$^ Patients could be diagnosed with more than one rheumatic diseases

^∞^ P-value from Fisher’s Exact test unless a=Mann Whitney U test
