# Supplementary material for: Predictors of hospitalization in patients with rheumatic disease and COVID-19 in Ireland: data from the COVID-19 global rheumatology alliance registry
Source: Rheumatol Adv Pract. 2021 May 13;5(2):rkab031. doi: 10.1093/rap/rkab031 (PMC8244588; doi:10.1093/rap/rkab031)
Supplement: rkab031_Supplementary_Data [file rkab031_supplementary_data.zip › 2021-039 Supplementary Material GRA members.docx]

| **Name** | **Institution/Affiliation** | **Country/Region** |
| --- | --- | --- |
| Brahim Dahou | Association Rhumatologues Algériens Privés (ARAP) | Algeria |
| Eva Rath | Hanusch Krankenhaus, Vienna | Austria |
| Yves Piette | AZ Sint-Jan Brugge | Belgium |
| Mieke Devinck | AZ Sint-Lucas Brugge | Belgium |
| Bea Maeyaert | AZ Sint-Lucas Brugge | Belgium |
| Francinne Machado Ribeiro | Hospital Universitário Pedro Ernesto Universidade do Estado do Rio de Janeiro | Brazil |
| Sandra Lucia Euzebio Ribeiro | Federal University of Amazonas | Brazil |
| Marcelo Pinheiro | Universidade Federal De São Paulo Escola Paulista de Medicina e Escola Paulista de Enfermagem | Brazil |
| Rosana Quintana | . | Brazil |
| Gimena Gómez | . | Brazil |
| Karen Roberts | . | Brazil |
| Roberto Miguel Baez | . | Brazil |
| Vanessa Castro Coello | . | Brazil |
| María J. Haye Salinas | . | Brazil |
| Federico Nicolas Maldonado | . | Brazil |
| Alvaro Andres Reyes Torres | . | Brazil |
| Gelsomina Alle | . | Brazil |
| Romina Tanten | . | Brazil |
| Hernán Maldonado Ficco | . | Brazil |
| Romina Nieto | . | Brazil |
| Carla Gobbi | . | Brazil |
| Yohana Tissera | . | Brazil |
| Cecilia Pisoni | . | Brazil |
| Alba Paula | . | Brazil |
| Juan Alejandro Albiero | . | Brazil |
| Maria Marcela Schmid | . | Brazil |
| Micaela Cosatti | . | Brazil |
| Maria Julieta Gamba | . | Brazil |
| Carlevaris Leandro | . | Brazil |
| María Alejandra Cusa | . | Brazil |
| Noelia German | . | Brazil |
| Veronica Bellomio | . | Brazil |
| Lorena Takashima | . | Brazil |
| Mariana Pera | . | Brazil |
| Karina Cogo | . | Brazil |
| Maria Soledad Gálvez Elkin | . | Brazil |
| María Alejandra Medina | . | Brazil |
| Veronica Savio | . | Brazil |
| Ivana Romina Rojas Tessel | . | Brazil |
| Rodolfo Perez Alamino | . | Brazil |
| Marina Laura Werner | . | Brazil |
| Sofía Ornella | . | Brazil |
| Luciana Casalla | . | Brazil |
| Maria de la Vega | . | Brazil |
| María Severina | . | Brazil |
| Mercedes García | . | Brazil |
| Luciana Gonzalez Lucero | . | Brazil |
| Cecilia Romeo | . | Brazil |
| Sebastián Moyano | . | Brazil |
| Tatiana Barbich | . | Brazil |
| Ana Bertoli | . | Brazil |
| Andrea Baños | . | Brazil |
| Sandra Petruzzelli | . | Brazil |
| Carla Matellan | . | Brazil |
| Silvana Conti | . | Brazil |
| Ma. Alicia Lazaro | . | Brazil |
| Gustavo Fabián Rodriguez Gil | . | Brazil |
| Fabian Risueño | . | Brazil |
| Maria Isabel Quaglia | . | Brazil |
| Julia Scafati | . | Brazil |
| Natalia Lili Cuchiaro | . | Brazil |
| Jonathan Eliseo Rebak | . | Brazil |
| Susana Isabel Pineda | . | Brazil |
| María Elena Calvo | . | Brazil |
| Eugenia Picco | . | Brazil |
| Josefina Gallino Yanzi | . | Brazil |
| Pablo Maid | . | Brazil |
| Debora Guaglianone | . | Brazil |
| Julieta Silvana Morbiducci | . | Brazil |
| Sabrina Porta | . | Brazil |
| Natalia Herscovich | . | Brazil |
| José Luis Velasco Zamora | . | Brazil |
| Boris Kisluk | . | Brazil |
| Maria Sol Castaños Menescardi | . | Brazil |
| Rosana Gallo | . | Brazil |
| María Victoria Martire | . | Brazil |
| Carla Maldini | . | Brazil |
| Cecilia Goizueta | . | Brazil |
| sabrina solange de la vega fernandez | . | Brazil |
| Carolina Aeschlimann | . | Brazil |
| Gisela Subils | . | Brazil |
| Sebastián Ibáñez | Clínica Alemana de Santiago | Chile |
| Anne-Marie Chassin-Trubert | Complejo Hospitalario San José | Chile |
| Lingli Dong | Tongji Hospital | China |
| Lui Cajas | Clinica Universitaria Colombia - Centro Medico Providencia Sanitas | Colombia |
| Marko Barešić | University Hospital Center Zagreb | Croatia |
| Branimir Anić | Div Clin Immunol Rheumatol; Dept Int Med, School of Med Zagreb, University Hospital Center Zagreb | Croatia |
| Melanie-Ivana Čulo | University Hospital Dubrava, Zagreb | Croatia |
| Tea Ahel Pavelić | Clinical Hospital Center Rijeka | Croatia |
| Kristina Kovačević Stranski | University hospital Osijek | Croatia |
| Boris Karanovic | UHC Zagreb | Croatia |
| Jiri Vencovsky | Institute of Rheumatology, Prague | Czechia |
| Marta Píchová | Medipont plus s.ro. , České Budějovice | Czechia |
| Maria Filkova | Institute of Rheumatology, Prague | Czechia |
| Hesham Hamoud | Al Azhar University Hospitals | Egypt |
| Dimitrios Vassilopoulos | Hippokration General Hospital, Athens | Greece |
| Gabriela Maria Guzman Melgar | Hospital del Valle, Honduras | Honduras |
| Ho So | Chinese University of Hong Kong | Hong Kong |
| Márta Király | Petz Aladár University Teaching Hospital, Győr | Hungary |
| Mahdi Vojdanian | Iran Rheumatology Center | Iran |
| Alexandra Balbir-Gurman | Rambam Rheumatology Institute, Haifa | Israel |
| Fatemah Abutiban | Kuwait Rheumatology Association | Kuwait |
| Julija Zepa | Pauls Stradins Clinical University Hospital, Riga | Latvia |
| Inita Bulina | Pauls Stradins Clinical University hospital, Riga | Latvia |
| Loreta Bukauskiene | Klaipeda university hospital | Lithuania |
| Beatriz Zaueta | Centro Medico del Angel | Mexico |
| Angel Alejandro Castillo Ortiz | Centro Medico Las Americas | Mexico |
| Erick Zamora Tehozol | Centro Medico Pensiones | Mexico |
| David Vega | Hospital General de Zona #17 | Mexico |
| Diana Cervántes Rosete | Instituto Nacional de Ciencias Médicas y Nutrición Salvador Zubirán | Mexico |
| Eduardo Martín Nares | Instituto Nacional de Ciencias Médicas y Nutrición Salvador Zubirán | Mexico |
| Tatiana Sofia Rodriguez-Reyna | Instituto Nacional de Ciencias Médicas y Nutrición Salvador Zubirán | Mexico |
| Marina Rull Gabayet | Instituto Nacional de Ciencias Médicas y Nutrición Salvador Zubirán | Mexico |
| Deshiré Alpízar-Rodríguez | Mexican College of Rheumatology | Mexico |
| Fedra Irazoque | Private Practice | Mexico |
| Xochitl Jimenez | Centro Medico Naval | Mexico |
| Lenny Geurts-van Bon | Ziekenhuisgroep Twente | Netherlands |
| Theo Zijlstra | Isala Hospital, Zwolle | Netherlands |
| Monique Hoekstra | Isala Hospital, Zwolle | Netherlands |
| Nasra Al-Adhoubi | Royal Hospital | Oman |
| Babur Salim | Fauji Foundation Hospital | Pakistan |
| Enrique Giraldo | Complejo Hospitalario | Panama |
| Ariel Salinas | Hospital Essalud Alberto Sabogal Sologuren | Peru |
| Manuel Ugarte-Gil | Universidad Científica del Sur-Hospital Guillermo Almenara Irigoyen | Peru |
| Jarosław Nowakowski | University Hospital, Krakow | Poland |
| Samar Al-Emadi | Hamad Medical Corporation | Qatar |
| Richard Conway | St James' Hospital, Dublin | Republic of Ireland |
| Rachael Flood | Tallaght University Hospital | Republic of Ireland |
| Geraldine McCarthy | Mater Misericordiae University Hospital | Republic of Ireland |
| Ioana Felea | County Emergency Hospital, Cluj Napoca | Romania |
| Ileana Filipescu | County Emergency Hospital, Cluj Napoca | Romania |
| Simona Rednic | County Emergency Hospital, Cluj Napoca | Romania |
| Laura Groseanu | Sf Maria Clinical Hospital, Bucharest | Romania |
| Maria Magdelena Tamas | County Emergency Hospital, Cluj Napoca | Romania |
| Vanda Mlynarikova | National Institute of Rheumatic Diseases, Piešťany | Slovak Republic |
| Martina Skamlova | FNSPFDR, Banská Bystrica | Slovak Republic |
| Martin Zlnay | National Institute of Rheumatic Diseases, Piešťany | Slovak Republic |
| Dagmar Mičeková | National Institute of Rheumatic Diseases, Piešťany | Slovak Republic |
| Lubica Capova | University Hospital, Bratislava | Slovak Republic |
| Zelmira Macejova | University Hospital, Košice | Slovak Republic |
| Emőke Šteňová | University Hospital Bratislava | Slovak Republic |
| Helena Raffayova | National Institute of Rheumatic Diseases, Piešťany | Slovak Republic |
| Gabriela Belakova | Medman s.r.o., Martin | Slovak Republic |
| Eva Strakova | Faculty hospital Prešov | Slovak Republic |
| Marieta Senčarová | Louis Pasteur University Hospital, Košice | Slovak Republic |
| Soňa Žlnayová | Poliklinika MarMedico, s.r.o., Nové Mesto nad Váhom | Slovak Republic |
| Anna Sabová | súkromná reumatologická ambulancia, Vranov nad Topľou | Slovak Republic |
| Daniela Spisakova | University Hospital od L. Pasteur Kosice | Slovak Republic |
| Mária Oetterová | Safarik University hospital, Kosice | Slovak Republic |
| Olga Lukacova | National Institute of Rheumatic Diseases, Piešťany | Slovak Republic |
| Martina Bakosova | UNB Nemocnica Stare Mesto, Bratislava | Slovak Republic |
| Alojzija Hocevar | UMC Ljubljana | Slovenia |
| Natalia de la Torre-Rubio | Hospital Universitario Puerta de Hierro Majadahonda | Spain |
| Juan José Alegre Sancho | Hospital Universitari Dr Peset, Valencia | Spain |
| Montserrat Corteguera Coro | Complejo Asistencial Avila | Spain |
| Juan Carlos Cobeta Garcia | Hospital Ernest Lluch, Calatayud | Spain |
| Maria Carmen Torres Martin | Hospital Nuestra Senora Sonsoles, Avila | Spain |
| Jose Campos | Hospital Universitario Puerta de Hierro | Spain |
| Jose A Gomez Puerta | Hospital Clinic Barcelona | Spain |
| Gozd Kubra Yardımcı | Hacettepe University Faculty of Medicine, Ankara | Turkey |
| Servet Akar | Izmir Katip Celebi University Atatürk Training and Research Hospital, Izmir | Turkey |
| Ozan Cemal Icacan | Bakırköy Dr. Sadi Konuk Research And Training Hospital, Istanbul | Turkey |
| Selda ÇELİK | BAKIRKOY DR SADI KONUK EDUCATIONAL AND RESEARCH HOSPITAL, RHEUMATOLOGY DEPARTMENT, Istanbul | Turkey |
| Viktoriia Vasylets | Multifield Medical Centre, Odessa | Ukraine |
| Su-Ann Yeoh | University College London Hospital, London | United Kingdom |
| Claire Vandevelde | Leeds Teaching Hospitals NHS Trust | United Kingdom |
| Sasha Dunt | Countess of Chester NHS Foundation Trust | United Kingdom |
| Jane Leeder | Norfolk & Norwich University Hospital | United Kingdom |
| Elizabeth Macphie | Lancashire and South Cumbria NHS Foundation Trust | United Kingdom |
| Rosaria Salerno | King's College Hospital | United Kingdom |
| Christine Graver | Hampshire Hospitals NHS Trust | United Kingdom |
| Katie Williams | York District Hospital | United Kingdom |
| Sheila O'Reilly | Royal Derby Hospital | United Kingdom |
| Kirsty Devine | York/Scarborough Hospitals | United Kingdom |
| Jennifer Tyler | Royal United Hospital, Bath | United Kingdom |
| Elizabeth Warner | Lister Hospital | United Kingdom |
| James Pilcher | University Hospital Lewisham | United Kingdom |
| Samir Patel | Queen Elizabeth hospital Woolwich | United Kingdom |
| Elena Nikiphorou | King's College Hospital | United Kingdom |
| Laura Chadwick | St Helens & Knowsley NHS Foundation Trust | United Kingdom |
| Caroline Mulvaney Jones | Llandudno Hospital | United Kingdom |
| Beverley Harrison | Salford Royal NHS FT | United Kingdom |
| Lucy Thornton | Bradford Royal Infirmary | United Kingdom |
| Diana O'Kane | RNHRD at Royal United Hospital Bath | United Kingdom |
| Lucia Fusi | King's College Hospital | United Kingdom |
| Audrey Low | Salford Royal NHS FT | United Kingdom |
| Sarah Horton | Minerva Health Centre | United Kingdom |
| Shraddha Jatwani | Albert Einstein Medical Center, PA | United States of America |
| Sara Baig | Arthritis and Rheumatology Consultants, PA | United States of America |
| Hammad Bajwa | Arthritis and Rheumatology Consultants, PA | United States of America |
| Vernon Berglund | Arthritis and Rheumatology Consultants, PA | United States of America |
| Angela Dahle | Arthritis and Rheumatology Consultants, PA | United States of America |
| Walter Dorman | Arthritis and Rheumatology Consultants, PA | United States of America |
| Jody Hargrove | Arthritis and Rheumatology Consultants, PA | United States of America |
| Maren Hilton | Arthritis and Rheumatology Consultants, PA | United States of America |
| Nicholas Lebedoff | Arthritis and Rheumatology Consultants, PA | United States of America |
| Susan Leonard | Arthritis and Rheumatology Consultants, PA | United States of America |
| Jennifer Morgan | Arthritis and Rheumatology Consultants, PA | United States of America |
| Emily Pfeifer | Arthritis and Rheumatology Consultants, PA | United States of America |
| Archibald Skemp | Arthritis and Rheumatology Consultants, PA | United States of America |
| Jeffrey Wilson | Arthritis and Rheumatology Consultants, PA | United States of America |
| Anne Wolff | Arthritis and Rheumatology Consultants, PA | United States of America |
| Eduardo Cepeda | Austin Diagnostic Clinic | United States of America |
| Kristin D'Silva | Brigham and Women's Hospital | United States of America |
| Tiffany Hsu | Brigham and Women's Hospital | United States of America |
| Naomi Serling-Boyd | Brigham and Women's Hospital | United States of America |
| Jeffrey Sparks | Brigham and Women's Hospital | United States of America |
| Derrick Todd | Brigham and Women's Hospital | United States of America |
| Zachary Wallace | Brigham and Women's Hospital | United States of America |
| Denise Hare | Capital Health Rheumatology | United States of America |
| Cassandra Calabrese | Cleveland Clinic | United States of America |
| Christopher Adams | East Alabama Medical Center | United States of America |
| Arezou Khosroshahi | Emory University | United States of America |
| Adam Kilian | George Washington University | United States of America |
| Douglas White | Gundersen Health System | United States of America |
| Melanie Winter | Gundersen Health System | United States of America |
| Theodore Fields | Hospital for Special Surgery | United States of America |
| Caroline Siegel | Hospital for Special Surgery | United States of America |
| Nicole Daver | Institute of Rheumatic and Autoimmune Diseases | United States of America |
| Melissa Harvey | Institute of Rheumatic and Autoimmune Diseases | United States of America |
| Neil Kramer | Institute of Rheumatic and Autoimmune Diseases | United States of America |
| Concetta Lamore | Institute of Rheumatic and Autoimmune Diseases | United States of America |
| Suneya Hogarty | Integrative Arthritis and Pain Consultants | United States of America |
| Karen Yeter | Kaiser Permanente | United States of America |
| Leanna Wise | Los Angeles County + USC Medical Center | United States of America |
| Faizah Siddique | Loyola University Medical Center | United States of America |
| Byung Ban | Medstar Georgetown University Hospital | United States of America |
| Tamar Tanner | Montefiore Medical Center | United States of America |
| Eric Ruderman | Northwestern Memorial | United States of America |
| William Davis | Ochsner Medical Center Rheumatology Department | United States of America |
| Robert Quinet | Ochsner Medical Center Rheumatology Department | United States of America |
| Evangeline Scopelitis | Ochsner Medical Center Rheumatology Department | United States of America |
| Karen Toribio Toribio | Ochsner Medical Center Rheumatology Department | United States of America |
| Tameka Webb-Detiege | Ochsner Medical Center Rheumatology Department | United States of America |
| Jerald Zakem | Ochsner Medical Center Rheumatology Department | United States of America |
| Khurram Abbass | Private Practice | United States of America |
| Gilbert Kepecs | Private Practice | United States of America |
| Lilliam Miranda | Rheumatology Center INC | United States of America |
| Michael Guma | Riverside Medical Group | United States of America |
| Ammar Haikal | Riverside Medical Group | United States of America |
| Sushama Mody | Riverside Medical Group | United States of America |
| Daric Mueller | Shores Rheumatology PC | United States of America |
| Arundathi Jayatilleke | Temple University Hospital | United States of America |
| JoAnn Zell | University of Colorado | United States of America |
| Alison Bays | University of Washington, Seattle | United States of America |
| Kathryn Dao | UT Southwestern Medical Center | United States of America |
| Ezzati Fatemeh | UT Southwestern Medical Center | United States of America |
| Deborah Parks | Washington University Div of Rheumatology | United States of America |
| David Karp | UT Southwestern Medical Center | United States of America |
| Guillermo Quiceno | UT Southwestern Medical Center | United States of America |
